# Unbiased Characterization of the Microbiome and Virome of Questing Ticks

**DOI:** 10.3389/fmicb.2021.627327

**Published:** 2021-05-12

**Authors:** Shona Chandra, Erin Harvey, David Emery, Edward C. Holmes, Jan Šlapeta

**Affiliations:** ^1^Sydney School of Veterinary Science, Faculty of Science, The University of Sydney, Sydney, NSW, Australia; ^2^Marie Bashir Institute for Infectious Diseases and Biosecurity, School of Life and Environmental Sciences and School of Medical Sciences, The University of Sydney, Sydney, NSW, Australia

**Keywords:** *cox*1, *Ixodes holocyclus*, microbiome, paralysis tick, virome, 16S rRNA, NSW, bacterial profile

## Abstract

Due to their vector capacity, ticks are ectoparasites of medical and veterinary significance. Modern sequencing tools have facilitated tick-associated microbiota studies, but these have largely focused on bacterial pathogens and symbionts. By combining 16S rRNA gene sequencing with total RNA-sequencing methods, we aimed to determine the complete microbiome and virome of questing, female *Ixodes holocyclus* recovered from coastal, north-eastern New South Wales (NSW), Australia. We present, for the first time, a robust and unbiased method for the identification of novel microbes in ticks that enabled us to identify bacteria, viruses, fungi and eukaryotic pathogens. The dominant bacterial endosymbionts were *Candidatus* Midichloria sp. Ixholo1 and *Candidatus* Midichloria sp. Ixholo2. *Candidatus* Neoehrlichia australis and *Candidatus* Neoehrlichia arcana were also recovered, confirming that these bacteria encompass *I. holocyclus*’ core microbiota. In addition, seven virus species were detected—four previously identified in *I. holocyclus* and three novel species. Notably, one of the four previously identified virus species has pathogenic potential based on its phylogenetic relationship to other tick-associated pathogens. No known pathogenic eukaryotes or fungi were identified. This study has revealed the microbiome and virome of female *I. holocyclus* from the environment in north-eastern NSW. We propose that future tick microbiome and virome studies utilize equivalent methods to provide an improved representation of the microbial diversity in ticks globally.

## Introduction

The study of microorganisms associated with ticks (Acari: *Ixodidae*) has gained global attention as factors such as climate change and increasing urbanization have driven an increase in cases of tick-borne disease (Levi et al., 2015; Jia et al., 2020). Following mosquitos, ticks are arguably the second most important arthropod disease vector, owing to their ability to harbor a diversity of microorganisms simultaneously including bacteria, viruses, fungi, and eukaryotic parasites (de La Fuente et al., 2008; Brites-Neto et al., 2015). Elements of tick biology, such as their distinct life cycle stages, tendency to feed on multiple hosts over their lifetime and comparatively long lifespan, make them unique and potent vectors (Sonenshine and Roe, 2013). Ticks are found on all continents and carry a diversity of pathogenic species affecting humans as well as domestic animals, most notably, *Borrelia burgdorferi* sensu lato (s.l.), the causative agent of Lyme borreliosis, which is found in North America, Europe, and Asia (Anderson, 1989; Bunikis et al., 2004). The study of tick-associated microorganisms has, to date, largely focused on bacterial pathogens, particularly *B. burgdorferi* s.l., with many tick microbiome studies utilizing targeted bacterial 16S ribosomal RNA (16S rRNA) gene sequencing (Pepin et al., 2012; Gofton et al., 2015b; Van Treuren et al., 2015; Venere et al., 2015; Thapa et al., 2019; Jia et al., 2020). While this research is vital to the understanding of these pathogens, it necessarily ignores large numbers of other potentially zoonotic pathogens. This is of concern in areas where the cause of tick-associated disease is unknown.

In Australia, the study of the microbial communities of ticks is of particular interest as cases of undiagnosed suspected tick-associated disease have been documented since the 1980s, particularly along the central eastern coastline (Stewart et al., 1982). It has remained a topic of contention in the following four decades whether *B. burgdorferi* s.l. is present in Australia and if the Australian paralysis tick, *Ixodes holocyclus* Neumann, 1899, is a competent vector (Piesman and Stone, 1991; Russell et al., 1994; Dehhaghi et al., 2019). To date, there has been no evidence supporting locally acquired *B. burgdorferi* s.l. infections as the spirochaete has not yet been found in Australia (Russell et al., 1994; Gofton et al., 2015a; Collignon et al., 2016; Chalada et al., 2018; Chandra and Šlapeta, 2020). Several studies have been undertaken in search of a causative agent for tick associated disease in the region, although these have largely utilized a 16S rRNA gene sequencing approach which limits pathogen discovery to bacterial species (Greay et al., 2018; Chandra and Šlapeta, 2020). In recent years, only two studies have explored the virome of Australian ticks, which have identified 20 novel RNA virus species, with one predicted to have pathogenic potential based on its phylogenetic relationship to other arboviruses in the *Coltivirus* genus (O’Brien et al., 2018; Harvey et al., 2019).

The field of microbiology has changed dramatically with the increased use of metagenomic sequencing technologies. Techniques such as 16S rRNA gene profiling have led to the discovery of an enormous diversity of bacterial species, particularly those for which culture methods had not yet been developed. This technique reduces the time and cost of characterizing the bacterial community within a given sample, enabling largescale microbiome studies (Kuske et al., 1997; Suau et al., 1999). Similarly, the use of RNA-sequencing (RNA-seq) has led to an explosion of RNA virus discoveries, leading to fundamental changes in our understanding of RNA virus diversity and evolution. Large scale virome studies have identified hundreds of novel virus species as well as novel genome organizations, as well as new virus families and orders (Cicuttin et al., 2015; Shi et al., 2015; Shi et al., 2016). These techniques are unbiased and require little to no prior knowledge of the microorganisms within a sample. As they become more widely used and the cost of sequencing decreases, metagenomic techniques have become a valuable method of identifying unknown causes of infectious disease. Indeed, in theory RNA-seq can be used to identify any infecting microorganism within a sample. Similarly, metagenomic methods have been used to identify novel pathogens both in humans and in animals (Edridge et al., 2019a, b; Chang et al., 2020).

Herein, we aimed to characterize the total microbiome and virome (i.e., the total assemblage of bacteria, parasites, fungi and viruses) of questing *I. holocyclus* from north-eastern NSW, Australia, with a particular focus on identifying known or potential pathogenic species responsible for cases of undiagnosed disease. Accordingly, we developed a comprehensive, unbiased approach to the characterization of the microbiome and virome of questing ticks, that enables identification of their “infectome.” Questing ticks were collected from fourteen localities and subjected to morphological and molecular identification to the species level. Metagenomic next-generation sequencing (mNGS) was then used to determine their 16S rRNA gene bacterial diversity profile at two hypervariable regions, and total RNA sequencing was performed on ribosomal (r)-RNA depleted samples from each location to identify previously characterized and novel viruses, as well as to identify fungi and eukaryotic parasites. The use of systematic and uniform tick collection across a defined region combined with the dual metagenomic approach enabled us to robustly characterize the complete microbiome and virome of *I. holocyclus* in this region.

## Materials and Methods

### Tick Specimens

**FIGURE 1:**
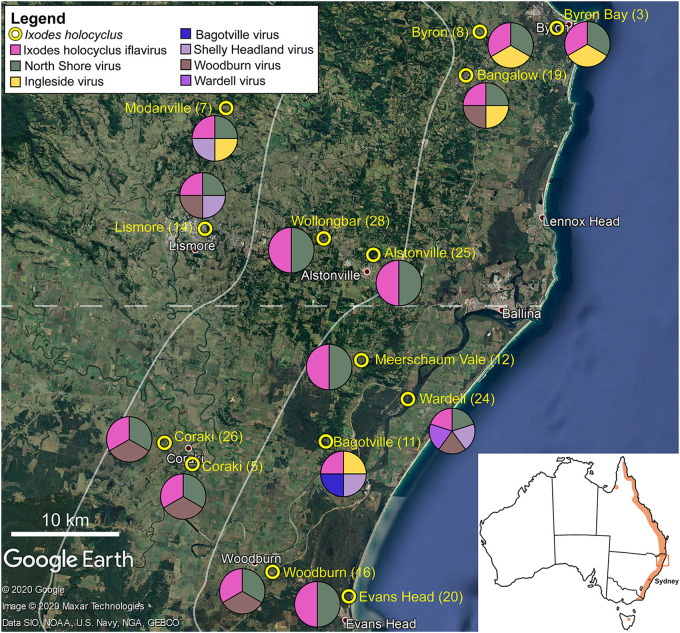
Representation of the approximate collection points for adult *Ixodes holocyclus* from the north-eastern coastline in NSW, Australia is shown. Tick sample number is provided in parentheses. The pie chart demonstrates the geographical spread of virus species identified in this study. Each circle is divided equally by the number of viruses found in the ticks collected at the corresponding site. Map of Australia with the approximate distribution of *I. holocyclus* highlighted (orange) along the eastern coastline in the subset. The map is sourced from data imported into Google Earth, 2020.

A total of 140 unfed adult female ticks were collected from 14 locations in north-eastern NSW, Australia (Figure 1). All ticks were collected from the environment and were stored alive in jars filled with soil matter and vegetation from the collection site. The ticks were stored at 12°C prior to morphological identification.

### Morphological and Molecular Identity of the Ticks for Microbial Analysis

The ticks were all morphologically identified using keys and guides (Roberts, 1970; Barker and Walker, 2014). Following morphological identification, the ticks were pooled into two sets of five ticks from each collection site. They were placed into sterile 1.0 mL CryoPure cryogenic vials (Sarstedt, Mawson Lakes, Australia) and stored at −80°C prior to DNA/RNA isolation.

The tick specimens for molecular confirmation and microbial analysis were surface sterilized prior to DNA isolation. Surface sterilization involved sequential 1 mL washes with a vortex for 1 min in 3% hydrogen peroxide (H_2_O_2_), two 30 s washes in 70% ethanol (w/v), 2 min in phosphate buffered saline (PBS, pH = 7.4) and dried on a Kimwipe (Kimberly-Clark, Ingleburn, Australia) (Swei and Kwan, 2017).

For molecular *cox*1 barcoding confirmation, one leg was removed at the fourth coxa for each tick (*n* = 70) using a new, sterile no. 11 scalpel blade and the genomic DNA (gDNA) was isolated in accordance with the ISOLATE II Genomic DNA Kit (Bioline, Eveleigh, Australia), with the following modifications: tick leg samples were digested in 180 μL of lysis buffer and 25 μL of proteinase K for 12–16 h in 56°C on a heat-block, and 80 μL of gDNA was eluted.

For the microbial analysis, gDNA was isolated from the pooled groups of five *I. holocyclus* (*n* = 14) for each collection site. The ticks were initially dissected into 1–2 mm pieces using new, sterile no. 11 scalpel blades, and then placed into a new 1.5 mL microcentrifuge tube for gDNA isolation using the ISOLATE II Genomic DNA Kit (Bioline, Eveleigh, Australia), with the following modifications: the tick pools were suspended in 180 μL of lysis buffer and 25 μL of proteinase K and were crushed using sterilized glass beads and new, sterilized polypropylene pestles (Sigma-Aldrich, Castle Hill, Australia). The homogenized tick pools were digested overnight for 12–16 h in 56°C on a heat-block, and 100 μL of gDNA was eluted. Two DNA extraction controls (blank reactions) were isolated alongside the pooled tick gDNA isolation to monitor for DNA isolation kit contamination.

### Amplification of the Tick Mitochondrially Encoded Cytochrome C Oxidase Subunit I (*cox*1) Gene

A 604 nucleotide (nt) 5′ fragment of the cytochrome c oxidase subunit I (*cox*1) was amplified using the following primer pairs: S0725 (5′-TAC TCT ACT AAT CAT AAA GAC ATT GG-3′) and S0726 (5′-CCT CCT CCT GAA GGG TCA AAA AAT GA-3′) (Kushimo, 2013).

MyTaq^TM^ Red Mix (Bioline, Eveleigh, Australia) was used for the *cox*1 amplification in 30 μL reactions with 2 μL of template DNA. The PCR cycling conditions were as follows: 95°C for 1 min, 35 cycles of 95°C for 15 s, 55°C for 15 s and 72°C for 10 s followed by 72°C for 5 min. All reactions contained a positive control with PCR-grade water used as a no template control. All conventional PCR reactions were conducted in a T100^TM^ Thermal Cycler (BioRad, Gladesville, Australia). PCR products of the individual tick leg gDNA (*n* = 70) were sequenced at Macrogen Ltd. (Seoul, South Korea).

### Tick DNA Sequence Analysis

Sequences were assembled using CLC Main Workbench 6.8.1 (Qiagen, Vedbæk, Denmark). Analysis of the composition of the nucleotide sequences and phylogenetic analysis of nymph tick DNA were performed using MEGA 7.0 (Kumar et al., 2016). Phylogenetic comparison was made between available *Ixodes* spp. haplotypes, with *Bothriocroton* spp. and *Haemaphysalis* spp. haplotypes from GenBank (National Centre for Biotechnology Information, NCBI) used as outgroups. This enabled us to determine the haplotypic diversity within the north-eastern NSW tick population (Kumar et al., 2016). Phylogenetic trees were inferred using both maximum likelihood (Tamura 3-parameter nucleotide model and gamma distribution of among-site rate variation with four categories; ML T92+Γ) and minimum evolution methods (Tamura-Nei model; ME TN93) available in MEGA 7.0 (Rzhetsky and Nei, 1992; Tamura, 1992; Tamura and Nei, 1993; Kumar et al., 2016).

### Amplification and Analysis of the Tick Bacterial Profile

Two target 16S rRNA hypervariable regions (V1–V3, V3–V4) were used to determine the bacterial diversity profile. The pooled tick gDNA samples (*n* = 28; V1–V3: 14, V3–V4: 14) and negative controls comprising blank reactions (*n* = 4; V1–V3: 2, V3–V4: 2) underwent diversity profiling of the 16S rRNA gene at the Australian Genome Research Facility (AGRF, Melbourne, Australia). DNA quality control (QC) screening via PCR was performed and indexing fluorometry at AGRF (Melbourne, Australia) prior to the diversity profiling at the 16S rRNA primer targets. All samples (*n* = 32) passed QC, including the blank reactions. Both V1–V3 and V3–V4 were sequenced on the Illumina MiSeq (300 nt paired end reads) using the following assays: 16S (V1–V3): 27F (5′-AGA GTT TGA TCM TGG CTC AG-3′) with 519R (5′-GWA TTA CCG CGG CKG CTG-3′) and 16S (V3–V4): 341F (5′-CCT AYG GGR BGC ASC AG-3′) with 806R (5′-GGA CTA CNN GGG TAT CTA AT-3′). Paired end reads were assembled using PEAR (version 0.9.5) and the primers were identified, trimmed and processed as previously described (Panetta et al., 2017).

### Multivariate Statistical Analysis for the Pooled Tick Microbiota

Multivariate statistical analyses were used to determine patterns of variation within the bacterial composition of the 5-tick pools of unfed *I. holocyclus* (*n* = 14) and two blank reactions. The microbiota abundance matrix, OTU taxonomy, and sample factors were imported as metadata for the multivariate analysis in PRIMER v.7 (PRIMER-e, Albany, New Zealand).

The data from the 16S rRNA V1–V3 (*n* = 16) and V3–V4 (*n* = 16) hypervariable regions were analyzed separately. Each tick sample was annotated with the following sample factors: tick species, DNA isolation kit, tick ID, sequence ID, collection location, orientation, and coastal proximity. Tick ID was the identification number associated with the pooled tick samples, while sequence ID was the identification number associated with the sequencing at AGRF (Melbourne, Australia). Collection location was recorded as the suburb in which the ticks were collected. Orientation reflected whether the tick was collected north or south of Ballina, NSW. Coastal proximity was determined by the distance from the coast based on the tick’s collection location (1: 0–10 km, 2: 10.1–20 km, 3: >20 km).

The V1–V3 assay yielded 1,903,906 paired end raw reads (1.15 Gb), quality filtered into 1,390,878 (min. 20,298; max. 22,109; *n* = 16) high quality reads excluding singletons and clustered into 473 bacterial OTUs. The V3–V4 assay yielded 1,717,029 paired end raw reads (1.03 Gb), quality filtered into 1,269,997 (min. 12,855; max. 146,582; *n* = 16) high quality reads excluding singletons and clustered into 278 bacterial OTUs. To clean the data, unassigned OTUs, mitochondria, and chloroplasts were excluded from the analyses. The total sum of the OTUs from all samples were obtained from the data matrix of OTUs and taxonomy abundance, and if the total sum of the reads within an OTU was <50 it was removed from analysis. OTUs present in the blank negative control reactions were removed, except for OTUs identified as *Ca*. Midichloria sp. Ixholo1 (OTU_1) or *Ca*. Midichloria sp. Ixholo2 (V1–V3: OTU_2; V3–V4: OTU_266) as they were important for microbial analysis. This resulted in 50 OTUs (1,283,130 paired end reads; *n* = 16) and 53 OTUs (1,225,035 paired end reads; *n* = 16) for the microbial analysis at the V1–V3 and V3–V4 regions, respectively.

The cleaned data was imported into PRIMER v.7 (PRIMER-e, Albany, New Zealand), and the data matrix was standardized (samples by total) and fourth root transformed. Bray-Curtis dissimilarity was used to measure the bacterial composition variation within the ticks. Non-metric multi-dimensional scaling ordination, nMDS (Kruskal and Wish, 1978), was used to view trends in bacterial community similarity among all samples. The goodness-of-fit from the two-dimensional nMDS plot was measured with Kruskal’s stress formula I (Kruskal and Wish, 1978). Visualization of the two-dimensional nMDS plots were enriched by annotating the different sample factors overlaid on to the ordination plots using symbols to assess their possible impacts on the composition of the bacterial communities. Analysis of similarities, ANOSIM (Clarke, 1993) (significance level, *p =* 0.05), was applied to test the null hypothesis of no difference between the bacterial communities.

The Mann-Whitney Test for significance was performed on Prism 8 (GraphPad Software, San Diego, United States of America) to determine whether the bacterial communities generated from the 16S rRNA hypervariable regions tested (V1–V3 and V3–V4) were significantly different from each other.

### RNA Extraction and Sequencing

Total RNA was extracted from ticks as described previously (Harvey et al., 2019). Briefly, individual ticks were washed in cold 100% ethanol and extracted individually using the Qiagen RNeasy Mini Kit (Chadstone, Australia). Each sample was then analyzed on the 2100 Bioanalyzer (Agilent, Mulgrave, Australia) to ensure the quality of the RNA sample and then pooled by collection location, with five ticks in each pool. Pooled samples were then subjected to rRNA depletion using the Ribo-Zero-Gold (epidemiology) kit (Illumina) and sequencing libraries were constructed using the TruSeq RNA Library Prep Kit (Illumina, Scoresby, Australia). Paired end sequencing was then performed for all libraries on the HiSeq2500 platform. All library preparation and sequencing were performed at AGRF (Melbourne, Australia).

### Virus Identification and Abundance Measure

RNA-seq reads were trimmed using Trimmomatic (v. 0.36) (Bolger et al., 2014) and *de novo* assembled into contigs using Trinity (v. 2.5.1) (Grabherr et al., 2011). Assembled reads were then blasted against the GenBank nr database (NCBI, March 2020) using diamond BLASTx (v. 0.9.25) (Buchfink et al., 2015). Contigs showing amino acid sequence similarity to viral proteins were checked for sequence similarity to non-viral sequences against the GenBank nt database (NCBI, March 2020) using BLASTn (BLAST+ 2.9.0) (Altschul et al., 1990). Where possible, contigs were further assembled into larger genome fragments manually in Geneious (v. 9.1.8) (Kearse et al., 2012). The abundance of assembled contigs or genomes were then measured using Bowtie2 (v. 2.2.5) (Langmead and Salzberg, 2012). rRNAs were removed from the total number of reads for abundance calculations by identifying rRNA contigs obtained through blasting the assembled contigs against the rRNA sequences available in the NCBI nt database (March 2020). The unassembled fastq files were then aligned to these sequences using Bowtie2 (v.2.2.5). Abundance graphs were produced by calculating RPKM (reads per kilobase million) using functions within the tidyverse package (v.1.3.0) in R (v.3.6.3).

### Phylogenetic Analysis of Viral Sequences

To determine the phylogenetic relationship of each virus to previously characterized viruses, the blast hit with the highest sequence similarity was used to identify the relevant virus family. The relatively well conserved RNA dependent RNA polymerase (RdRp) region or polyprotein amino acid sequences for the relevant families were extracted from the NCBI RefSeq database (March 2020) and a web-based Blast search was used to ensure all related sequences were included in the phylogenetic analysis. The recovered sequences were aligned with MAFFT (v. 7.300) (Katoh and Standley, 2013) using the L-INS-I algorithm. Sequence alignments were then trimmed to remove large gaps and expanses of unaligned sequences using TrimAL (v. 1.4.1) (Capella-Gutierrez et al., 2009). The resulting trimmed alignments were then manually checked to ensure their length and quality. This process resulted in five alignments of length equal to or greater than 415 amino acids. The maximum likelihood method in IQ-Tree (v. 1.6.12) (Nguyen et al., 2015) was then used to infer phylogenetic trees for each alignment. The model finder function (Kalyaanamoorthy et al., 2017) was enabled to select the best-fit model of nucleotide substitution for each alignment, and 1000 bootstrap replications were performed to assess the statistical support for each node.

### Screening for Fungal and Parasitic Pathogens

To identify pathogenic fungi or eukaryotic parasites within the RNA-seq data set, the fastq files were analyzed using ccMetagen (Marcelino et al., 2020) to taxonomically categorize the sequence alignment output produced by KMA (Clausen et al., 2018). The results were filtered to exclude hits to viruses, bacteria, and plant species by superkingdom classification. The output was then further filtered to include only those hits to species known to act as pathogens in mammals. The nucleotide sequence for the closest match to each hit was extracted from NCBI and the fastq reads aligned using Bowtie2 (v2.2.5) (Langmead and Salzberg, 2012) to these sequences to manually remove hits where reads aligned to low complexity regions.

## Results

### Female *Ixodes holocyclus* Ticks Recovered From North-Eastern NSW

Unfed adult *I. holocyclus* specimens (*n* = 140) were collected from vegetation across 14 locations in north-eastern NSW, Australia (Figure 1). For identification via molecular barcoding, five ticks from each collection site (14 sites, 70/140 ticks) were selected for the *cox*1 mitochondrial DNA amplification using DNA isolated from a single leg. Accordingly, we identified fourteen distinct *cox*1 haplotypes of *I. holocyclus* (*n* = 70; 98.94–100%, AB075955) randomly distributed across the sampled localities (Supplementary Figure 1).

### Presence of *Candidatus* Midichloria spp. in Ticks From North-Eastern NSW

We utilized two 16S rRNA gene diversity profiling assays—V1–V3 and V3–V4. The V1–V3 diversity profiling assay yielded 1,903,906 paired end raw reads (1.15 Gb), which were quality filtered into 1,390,878 (min. 20,298; max. 221,091; *n* = 16) high quality reads excluding singletons. The V3–V4 diversity profiling assay yielded 1,717,029 paired end raw reads (1.03 Gb) quality filtered into 1,269,997 (min. 12,855; max. 146,582; *n* = 16) high quality reads excluding singletons. Both hypervariable regions were dominated by OTUs belonging to *Candidatus* Midichloria sp. Ixholo1 (V1–V3: OTU_1, 79.7%; V3–V4: OTU_1, 76.5%) and *Candidatus* Midichloria sp. Ixholo2 (V1–V3: OTU_2, 19.4%; V3–V4: OTU_266, 19.1%), respectively. OTUs generated for the V1–V3 or V3–V4 hypervariable regions were not significantly different (Mann-Whitney test, two-tailed *t*-test; *p* = 0.624). When the two most common OTUs were excluded from analyses, the reads were dominated by the phyla Actinobacteria (V1–V3: 60.8%; V3–V4: 39.0%) and Proteobacteria (V1–V3: 39.2%; V3–V4: 53.1%).

**FIGURE 2:**
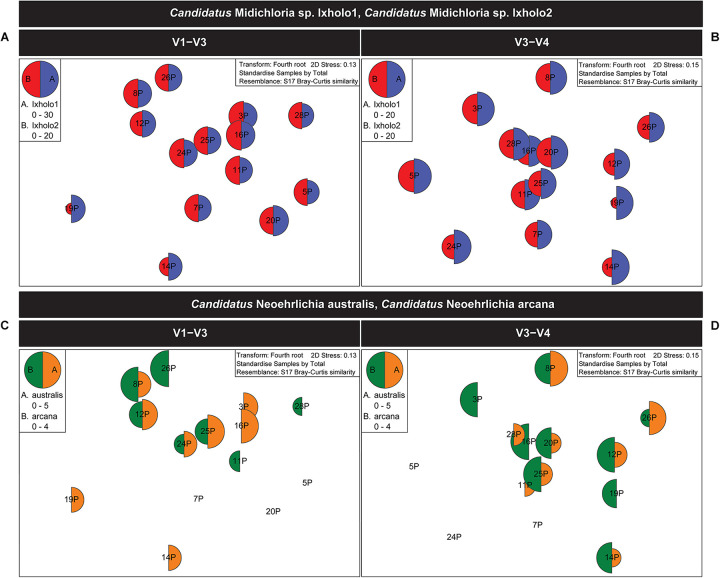
Multivariate statistical analysis of the V1–V3 and V3–V4 16S rRNA bacterial profile for the pooled samples of female *Ixodes holocyclus* from the far north-eastern region of NSW, Australia. Non-metric multidimensional scaling (nMDS) ordination plots for bacterial abundance of *Candidatus* Midichloria spp. and *Candidatus* Neoehrlichia spp. are shown. **(A,B)** nMDS ordination plots for bacterial abundance of *Ca*. Midichloria sp. Ixholo1 (blue) and *Ca*. Midichloria sp. Ixholo2 (red) revealed that these endosymbionts were prevalent across all pooled groups of ticks at the V1–V3 **(A)** and V3–V4 **(B)** hypervariable regions. **(C,D)** nMDS ordination plots for bacterial abundance of *Ca*. Neoehrlichia arcana (orange) and *Ca*. Neoehrlichia australis (green) revealed that these endosymbionts were mostly prevalent across the pooled groups of ticks at the V1–V3 **(C)** and V3–V4 **(D)** hypervariable regions. *Candidatus* Neoehrlichia spp. was not detected in samples 5P and 7P at both hypervariable regions, while sample 20P and 24P was not detected in the V1–V3 and V3–V4, respectively.

Unlike *Ca*. Midichloria sp. Ixholo1 and *Ca*. Midichloria sp. Ixholo2, the suspect pathogenic *Candidatus* Neoehrlichia australis and *Candidatus* Neoehrlichia arcana were not uniformly present in all pooled samples across both hypervariable regions (Figure 2).

### Permutation-Based Hypothesis Testing Reveals Orientation and Coastal Proximity Do Not Impact the Microbiota

Non-metric multi-dimensional scaling ordination (nMDS) (Kruskal and Wish, 1978), was used to view the trends of bacterial community similarity between all samples. The goodness-of-fit from the two-dimensional nMDS plot was measured with Kruskal’s stress formula I (Kruskal and Wish, 1978). The nMDS ordination plots for bacterial abundance at the OTU level appeared to reveal clustering for the factors, “orientation” and “coastal proximity,” in both hypervariable regions (Figure 2). The factor “orientation” was defined as whether the tick samples were collected north or south of Ballina, NSW, while for coastal proximity, we assigned each sample to a category (1–3) based on distance from the coast (1: 0–10 km, 2: 10.1–20 km, 3: >20 km) (Figure 1).

**FIGURE 3:**
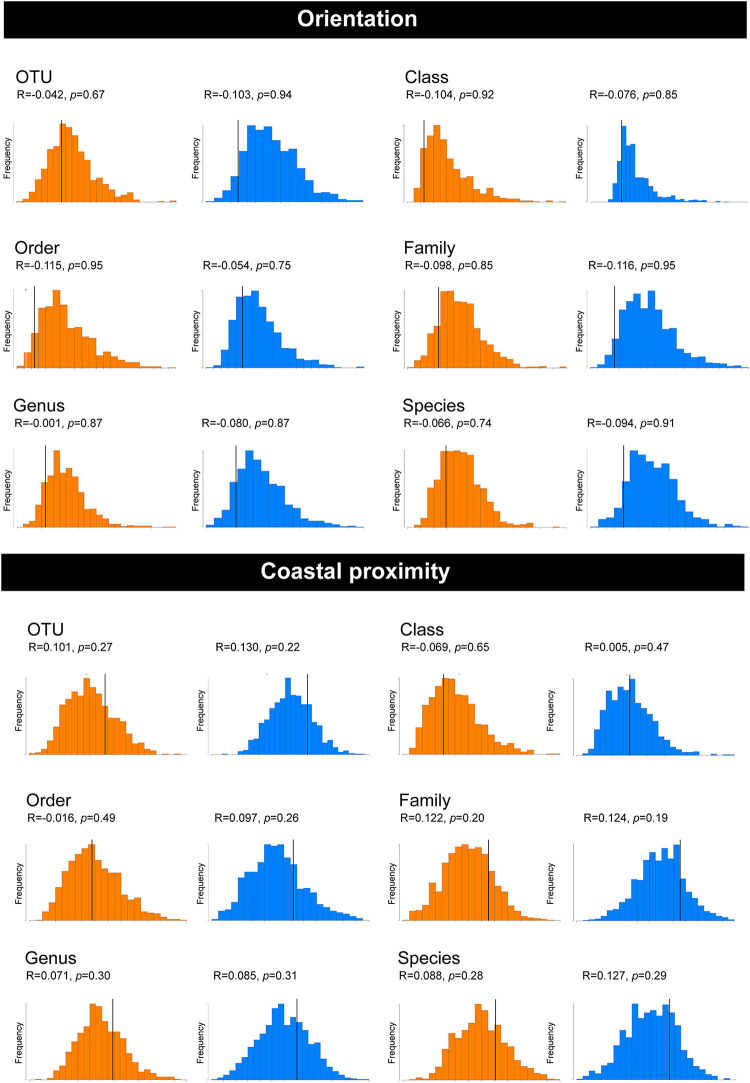
Analysis of similarities (ANOSIM) histograms for the pooled samples of female *Ixodes holocyclus* at the V1–V3 and V3–V4 16S rRNA hypervariable region. Histograms generated for the V1–V3 hypervariable region have been indicated in orange and the V3–V4 region has been indicated with blue histograms. ANOSIM histograms were generated for factors orientation and coastal proximity. The bacterial abundance has been compared at the following levels: OTU, Class, Order, Family, Genus, and Species for both factors.

To determine whether orientation and coastal proximity had a significant effect on the tick samples, we utilized permutation-based hypothesis testing approach (analysis of similarities, ANOSIM) on the Bray-Curtis dissimilarity matrix (Figure 3). Taking into account the north-south orientation as the external factor, and bacterial OTU, class, order, family, genus, and species, the ANOSIM histograms revealed no significant difference between the unordered “orientation” sub-groups (north, south) at either hypervariable region (Figure 3). Similarly, sub-groups based on the “coastal proximity” (1: 0–10 km, 2: 10.1–20 km, 3: >20 km) as the factor were not significant using ANOSIM at either hypervariable regions (Figure 3).

### RNA-Seq Virome Analysis of Unfed *I. holocyclus* in North-Eastern NSW

Unbiased RNA-sequencing was performed on a subset of five ticks from each collection location (*n* = 70) and subjected to an RNA virome analysis pipeline. Sequencing generated between 26,288,224 and 34,365,958 paired end reads for the 14 libraries which were subsequently trimmed and assembled *de novo* into between 140,430 and 325,716 contigs (Table 1).

**TABLE 1:** RNA-sequencing read numbers and assembled contigs.

**Location ID**	**Location name (NSW)**	**Number of reads**	**Number of contigs**
03	Byron Bay	32,787,426	325,716
05	Coraki	28,696,194	225,812
07	Modanville	26,718,102	250,555
08	Byron	26,867,404	195,169
11	Bagotville	30,367,208	246,149
12	Meerschaumvale	31,385,236	280,761
14	Lismore	26,288,224	140,430
16	Woodburn	30,338,554	281,398
19	Bangalow	32,077,798	232,777
20	Evans Head	27,660,994	233,635
24	Wardell	34,365,958	295,403
25	Alstonville	29,814,166	199,428
26	Coraki	28,766,056	288,448
28	Wollongbar	31,650,530	225,995

Complete or partial genomes of seven RNA viruses were identified within the 14 libraries, three of which were novel virus species and denoted Bagotville virus, Woodburn virus and Wardell virus after their sampling location of highest abundance (Table 2). The four previously identified viruses in the data set (Ingleside virus, *Ixodes holocyclus* iflavirus, Northshore virus, Shelly headland virus) were all *I. holocyclus*-associated and had been identified in *I. holocyclus* collected across the greater central east coast region of Australia (southern Queensland to southern NSW) (O’Brien et al., 2018; Harvey et al., 2019). The viruses identified here belonged to four defined virus families (*Iflaviridae*, *Partitiviridae*, *Reoviridae* and *Narnaviridae*) and two novel viruses clustered phylogenetically with groups of undefined clades sitting outside the *Phenuiviridae* and *Virgaviridae* families.

**TABLE 2:** Description of virus hits.

**Virus name**	**Closest hit**	**Genome organization**	**% identity**	**Family**	**Length (nt)**	**No. genome segments**	**Location IDs**
*Ixodes holocyclus* iflavirus	*Ixodes holocyclus* iflavirus	+ssRNA	98% (nt) 99.6% (aa)	*Iflaviridae*	9,262	1	03, 05, 07, 08, 11, 12, 14, 16, 19, 20, 24, 25, 26, 28
Northshore virus	Northshore virus	dsRNA	100% (nt)	*Partitiviridae*	1,405	1	03, 05, 07, 08, 11, 12, 14, 16, 19, 20, 24, 25, 26, 28
Shelly headland virus	Shelly headland virus	dsRNA		*Reoviridae*		11	07, 11, 14, 24
Ingleside virus	Ingleside virus	+ssRNA	99.8% (nt)	*Virgaviridae*	11,178	1	03, 07, 08, 19
Bagotville virus	Ribovira sp.	+ssRNA	82% (aa)	*Virgaviridae*-like	467	1	11
Woodburn virus	Beihai narna-like virus 21	+ssRNA	33.3% (aa)	*Narnaviridae*	1,668	1	05, 14, 16, 19, 24, 26
Wardell virus	Laurel lake virus	−ssRNA	75% (RdRp^*a*^)	*Phenuiviridae*	4,498	3	24

**^*a*^RNA-dependent RNA polymerase.**

*Ixodes holocyclus* iflavirus was identified in all 14 libraries and was the most abundant virus observed in each case except for library 19 (Figure 1). For example, in library 28 *Ixodes holocyclus* iflavirus constituted 2.2% of non-ribosomal reads, which is a relatively high abundance compared to the abundance of tick associated viruses observed in studies. All other viruses identified were at relatively low abundance. The abundance of virus reads varied substantially between libraries, although there was so association between viral load and location, nor was there any correlation between viral diversity and sampling location.

### Phylogenetic Analysis of the *I. holocyclus* Virome

**FIGURE 4:**
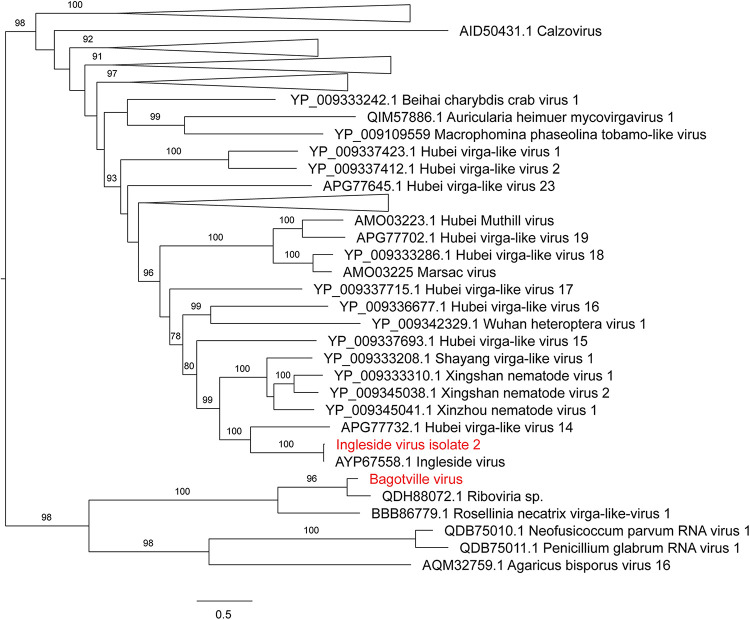
Maximum likelihood phylogenetic tree of *Virgaviridae*-like viruses. The tree is midpoint rooted for clarity only and uninformative branches have been collapsed. Bootstrap values of over 65% are shown. The names of virus sequences identified in this study are shown in red text.

The four viruses with a high degree of sequence similarity to previously identified virus sequences were considered to represent the same species. *Ixodes holocyclus* iflavirus and Northshore virus were identified in all 14 libraries, and each assembled genome showed approximately 98–99.9% nucleotide sequence similarity to the reference sequence (Supplementary Figure 2). Ingleside virus was identified in 4/14 libraries with a similar degree of sequence similarity (99.8%) in all four libraries (Figures 1, 4). Although levels of sequence similarity were similar across all libraries, there was some variation between the sequences in each library, suggesting that the presence of Ingleside virus in all libraries was not the result of contamination either before or during sequencing.

**FIGURE 5:**
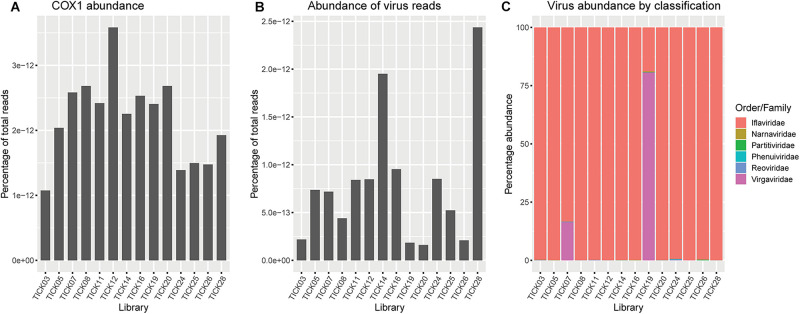
**(A)** Abundance of *Ixodes holocyclus cox*1 gene represented as reads per kilobase million (RPKM). **(B)** Abundance of total virus reads represented as RPKM. **(C)** Representation of the percentage of virus reads in each library broken down by family or the closest family where viruses do not cluster within a defined family. *Virgaviridae* and/or *iflaviridae* are highly abundant across all libraries, minimizing the representation of low abundance virus families, particularly *Phenuiviridae* and *Narnaviridae*.

The *Coltivirus* (family *Reoviridae*, double-strand RNA viruses), Shelly headland virus (SHLV) was detected at low abundance in 4 of the 14 libraries (Figures 1, 5). Partial genomes were assembled from each of the four positive libraries and it was determined that these represent genetic variants of the virus originally identified in feeding female *I. holocyclus* from Sydney, Australia. The nucleotide sequence identity for the RdRp region varied between 82 and 95% (Supplementary Figure 2), with the sequences identified in the southern coastal region showing greater similarity than those from the northern inland regions (Figure 1). The sequence similarity within the other 10 genome segments varied between ∼60 and 94% nucleotide sequence similarity.

**FIGURE 6:**
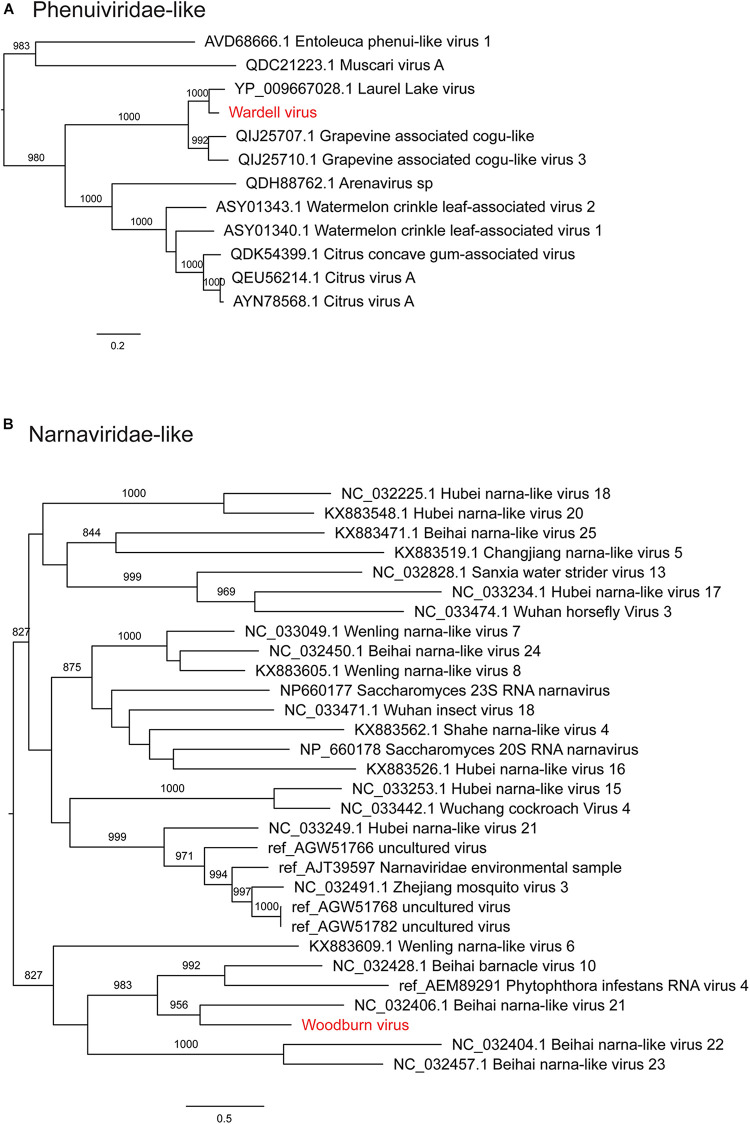
Maximum likelihood phylogenetic trees showing two novel virus sequences identified in this study from diverse families of RNA viruses. (**A**) Phenuiviridae and unclassified viruses clustering with this clade (Phenui-like viruses). (**B**) Narnaviridae and unclassified viruses clustering with this clade (Narna-like viruses). Trees are midpoint rooted for clarity and bootstrap values of over 65% are shown. The names of viruses identified in this study are shown in red text.

Notably, all three novel viruses identified clustered phylogenetically with viruses outside of currently classified virus families (Figures 4, 6). Bagotville virus clustered with a group of uncharacterized viruses that phylogenetically fall outside the *Virgaviridae* (single-strand positive-sense RNA viruses) that are normally associated with plants and fungi, showing the highest degree of similarity to an uncharacterized virus identified in soil (Figure 4). Woodburn virus clustered with *Narnaviridae*-like viruses (Figure 6; single-strand positive-sense RNA viruses), showing the highest degree of amino acid sequence similarity to a virus identified in sesarmid crab, although it is highly divergent from all sequences in NCBI (March 2020). Finally, Wardell virus clustered most closely with a recently identified tick associated virus identified in *Ixodes scapularis* Say, 1821 in North America, Laurel lake virus, and found within the *Phenuiviridae family* of single-strand negative-sense RNA viruses (Figure 6). Other viruses within this highly divergent clade are associated with plants or ticks.

### Analysis of Eukaryotic Parasites and Pathogens in RNA-seq Data

We used ccMetagen (Marcelino et al., 2020) to identify any putative eukaryotic pathogens that may be contained within each RNA-seq library. Accordingly, this analysis identified a number of species of fungi and eukaryotic microbial species. We reduced this list to 18 species previously associated with disease in mammals, eliminating arthropod infecting or environmental species (Supplementary Table 1). Of these 18 species, none are tick associated and most cause disease only in immunocompromised individuals (e.g., *Acremonium sclerotigenum*, *Roussoella solani*, *Conidiobolus lamprauges* etc.). Notably, however, we did identify the common brushtail possum-associated parasite, *Parastrongyloides trichosuri*, although this nematode parasite is neither tick associated nor linked with disease in humans (Mackerras, 1959).

## Discussion

We present a complete representation of the microbiome and virome of a tick species. As the cause of tick-borne disease in Australia remains unidentified, we propose a robust methodology for the characterization of the tick microbiome and virome based on tick sampling strategy and their biology. Our analysis identified known tick associated bacterial species, three novel virus species, and four virus species previously associated with *I. holocyclus*. Notably, one of the previously described viruses may have pathogenic potential in mammals due to its phylogenetic clustering within the *Coltivirus* genus that contains other disease associated viruses known or suspected to be transmitted by ticks.

In Australia, only a small number of metagenomic studies utilizing 16S rRNA gene targeted amplicon sequencing studies have been undertaken to elucidate the bacterial proponent of the tick microbiome and virome (Gofton et al., 2015a; Panetta et al., 2017; Chandra and Šlapeta, 2020). Similarly, only two studies have applied metagenomic technologies to determine the viral profile, or virome, of Australian ticks (O’Brien et al., 2018; Harvey et al., 2019). We present a methodology that provided the most comprehensive coverage of the microbiome and virome in female adult *I. holocyclus* from north-eastern NSW, Australia. Questing ticks were systematically collected across 14 locations representing varied environments in the north-eastern region of NSW. These ticks were kept alive at 12°C to become dormant, until being frozen at –80°C to ensure high quality RNA. It is important to note that questing ticks were used in this study as ticks are known to rarely take more than a single blood meal per lifecycle stage and deposit most of the acquired pathogen and endosymbionts into the host during the uptake of the blood meal (Nuttall et al., 1994; Nuttall and Labuda, 2003; Sonenshine and Roe, 2013). By analyzing only unfed *I. holocyclus* we assume that the microbial organisms identified within our study have the potential to be transmitted into the host, reducing the likelihood that the organism may be held solely within the ticks’ blood meal as was noted in the previous virome study of *I. holocyclus* (Harvey et al., 2019).

Two 16S rRNA gene amplicon profiles, V1–V3 and V3–V4, were analyzed for the bacterial profile. Panetta et al. (2017) determined that the amplification of the V1–V3 hypervariable region gave the greatest capacity to detect *Borrelia* spp. in samples of engorged female *Bothriocroton undatum* Fabricius, 1775. Despite this, the V3–V4 hypervariable region is preferred for the study of the bacterial profile in ticks as it has the highest estimated diversity among the sequenced bacterial communities (Sperling et al., 2017). To ascertain the most complete and rigorous bacterial profile for *I. holocyclus* from NE NSW, Australia, we determined that a combination of two bacterial profiling assays would be preferable.

We detected *Candidatus* Neoehrlichia arcana and *Candidatus* Neoehrlichia australis from female *I. holocyclus*. Both *Ca.* Neoehrlichia arcana and *Ca*. Neoehrlichia australis are closely related to *Candidatus* Neoehrlichia mikurensis, the causative agent of human Neoehrlichiosis (Gofton et al., 2016; Silaghi et al., 2016). Neoerhlichiosis is a poorly understood systemic inflammatory syndrome primarily seen in Europe and associated with *I. ricinus* and small rodents (Silaghi et al., 2016). The prevalence of *Ca.* Neoehrlichia mikurensis in *I. ricinus* varies between 0 and 18%, at an average of ∼6.5% (Silaghi et al., 2016; Larsson et al., 2018; Jenkins et al., 2019). We recovered *Ca. Neoehrlichia* spp. from 78.6% (11/14) of the pools of unfed female *I. holocyclus* collected from the environment at two hypervariable regions. Previous studies have identified *Ca.* Neoehrlichia australis and *Ca.* Neoehrlichia arcana in *I. holocyclus* and one *Ixodes trichosuri* Roberts, 1960 (Gofton et al., 2015a; Gofton et al., 2016; Chandra and Šlapeta, 2020). The presence of the endosymbiont *Ca.* Neoehrlichia spp. in this study strongly supports their standing in the core microbiota of *I. holocyclus*. Chandra and Šlapeta (2020), detected *Ca.* Neoehrlichia spp. in nymph ticks only, and noted that *Ca*. Neoehrlichia spp. was absent in engorged male and female *I. holocyclus* collected from dogs. It has previously been determined that the adult tick microbiota is comprised predominantly of endosymbionts (Chicana et al., 2019). From this, we can assume that the *Ca*. Neoehrlichia spp. are potentially migrating out of the adult tick and into the host species, hence accounting for its absence in the engorged adult ticks (Chandra and Šlapeta, 2020).

The bacterial component of ticks contains endosymbionts and comprises the core microbiota associated with the tick species (Budachetri et al., 2018). The core microbiota comprises the known microbial symbionts that may have a biological or ecological functional role, and is unique to each tick species, but common among ticks of that species regardless of location (Narasimhan and Fikrig, 2015; Budachetri et al., 2018; Chicana et al., 2019). Among *Ixodes ricinus* Linnaeus, 1758, the core microbiota includes *Candidatus* Midichloria mitochondrii (CMM), which likely has a biological role (Lo et al., 2006; Sassera et al., 2006; Venere et al., 2015; Guizzo et al., 2020). In Australia, *Ca.* Midichloria spp. Ixholo1 and *Ca.* Midichloria spp. Ixholo2 dominate the bacterial profiles of *I. holocyclus* (Beninati et al., 2009; Gofton et al., 2015b; Chandra and Šlapeta, 2020). These *Ca*. Midichloria spp. are part of the core microbiota of *I. holocyclus* and like CMM, potentially has a biological function.

Previous studies of the *I. holocyclus* virome identified a diversity of RNA virus species, many of which were novel and highly divergent from the most closely related virus species. Here, we identified four of these viruses - *Ixodes holocyclus* iflavirus, Shelly headland virus, Ingleside virus and Northshore virus (O’Brien et al., 2018; Harvey et al., 2019). *Ixodes holocyclus* iflavirus and Northshore virus were identified in all 14 libraries, suggesting that they are commonly associated with *I. holocyclus*. Of these four viruses, Shelly headland virus (SHLV) is the only species with any phylogenetic relationship to a tick-borne pathogen of humans. The virus was detected at varying abundance in four of the 14 libraries and although the RdRp region was genetically similar to previously determined isolates of this virus, the other ten segments showed varying degrees of divergence (60–96% nucleotide sequence identity) from the reference sequences. Although these segments are known to have high variability in within coltiviruses, it is likely that the incomplete sequencing of these segments also contributes to the high levels of diversity observed. Importantly, the genus *Coltivirus* contains the tick-borne pathogen, Colorado tick fever virus, as well as other virus species identified in ticks and/or mammals. These include Lishui pangolin virus that is suspected of causing disease in pangolins although not yet identified in ticks (Gao et al., 2020). SHLV was previously identified in feeding ticks collected from long-nose bandicoots *Perameles nasuta* Geoffroy, 1804 in the North Sydney region of NSW, but as these ticks contained host blood, it was not known if the virus was associated with the tick or its marsupial host (Harvey et al., 2019). Here, we have demonstrated that *I. holocyclus* do, in fact, carry the virus. We propose that further research should be conducted to determine if SHLV has zoonotic potential.

We identified three novel virus species within the RNA-seq data, all of which were highly divergent and did not cluster phylogenetically within a distinct family group. Woodburn virus clustered within the *Narnaviridae-*like group of viruses, many of which were identified in fungal and protist species, and was most similar to Beihai narna-like virus 21 identified in Sesarmid crabs (Shi et al., 2016), although these viruses may in reality infect host associated symbionts. Bagotville virus clustered with a group of largely uncharacterized virus sequences most closely related to the *Virgaviridae*. The most closely related characterized virus, Rosellinia necatrix virga-like virus 1, is associated with fungi (Arjona-Lopez et al., 2018), and thus it is reasonable to predict that, like Woodburn virus, Bagotville virus is in fact associated with a fungal symbiont of the ticks sequenced here. Wardell virus was the only novel virus identified here to be closely related to a tick-associated virus species, Laurel lake virus, although it was still highly divergent (Tokarz et al., 2018). Laurel lake virus was identified in *I. scapularis* and clustered outside of known members of the order *Bunyavirales*, but has recently been classified by the ICTV as within the *Phenuiviridae* family. Although a number of tick associated *Bunyavirales* have been identified, Laurel lake virus is highly divergent from these viruses (Tokarz et al., 2018).

The RNA-seq data was further utilized to identify eukaryotic and fungal species with pathogenic potential. No known tick associated pathogens were identified in our data. A number of species with pathogenic potential were identified, although none of these are described as being tick associated and because metagenomics methods routinely identify microbes that are in fact component of animal diet and/or microbiomes, we cannot firmly associate these taxa with tick hosts. In addition, as sequencing depth and sensitivity increases, more contaminant microbial species are being identified in RNA-seq libraries, including viruses (Asplund et al., 2019). These contaminants may be introduced at all steps in RNA sequencing from sample collection through to sequencing. Indeed, although the ticks were washed with PBS prior to RNA extraction, it is possible that a number of microbial species may be superficially associated with the ticks as a result of their environment. Importantly, however, none of these species have been identified as common contaminants in RNA-sequencing.

In sum, this study represents a comprehensive metagenomic survey of unfed female *I. holocyclus* recovered from the environment across 14 localities in north-eastern coastal NSW, Australia, systematically characterizing the microbiome and virome of the most common tick species that attacks humans in this region. We confirmed that *Candidatus* Midichloria spp. Ixholo1, *Candidatus* Midichloria spp. Ixholo2, *Candidatus* Neoehrlichia arcana and *Candidatus* Neoehrlichia australis are part of the bacterial proponent of the core microbiota of *I. holocyclus*. In addition, we revealed a diversity of virus species similar to that seen in similar studies of *Ixodes* ticks, identifying three novel species of RNA viruses as well as a single previously identified virus with predicted pathogenic potential. No DNA viruses were identified in our study, consistent with previous virome studies in ticks. The method employed maximized the capacity to detect the many endosymbionts within *I. holocyclus*, as well as other bacterial and viral organisms of unknown pathogenicity. As there is a clear need for a more systematic approach to survey the tick microbiome and virome (Jia et al., 2020), we propose that future studies undertake a similar rigorous approach to characterizing the complete complement of tick microbiota to enable identification of the “infectome” of questing ticks.

## Data Availability Statement

Nucleotide sequence data from this study have been deposited to GenBank (NCBI), under the accession numbers MW012495–MW012564 for the unfed female *I. holocyclus cox*1 sequences. The 16S rRNA gene V1–V3 and V3–V4 bacterial profiles have been deposited to the Sequence Read Archive (SRA) database (NCBI) under the BioProject ID PRJNA664219 (https://www.ncbi.nlm.nih.gov/bioproject/PRJNA664219). PRIMER data files and other relevant files are available on LabArchives (https://doi.org/10.25833/m6w0-yt22). RNA-seq fastq files have been deposited to the SRA database (NCBI) under the BioProject ID PRJNA665124 (https://www.ncbi.nlm.nih.gov/bioproject/PRJNA665124). The nucleotide sequence data for the viruses from this study have been deposited to GenBank (NCBI), under the accession numbers MW741888–MW741896.

## Author Contributions

SC, EH, ECH, and JŠ conceptualized the study. SC, EH, ECH, and JŠ carried out the methodology and validation. SC and EH performed the formal analysis and investigation. DE, ECH, and JŠ obtained the resources. SC was responsible for data curation. SC and EH wrote the original draft. SC, EH, ECH and JŠ were involved in the review and editing of the manuscript. All authors contributed to the article and approved the submitted version.

## Conflict of Interest

The authors declare that the research was conducted in the absence of any commercial or financial relationships that could be construed as a potential conflict of interest.
