# Supplementary material for: Unbiased Characterization of the Microbiome and Virome of Questing Ticks
Source: Front Microbiol. 2021 May 12;12:627327. doi: 10.3389/fmicb.2021.627327 (PMC8153229; doi:10.3389/fmicb.2021.627327)
Supplement: Supplementary file 1 [file Table_1.DOCX]

Supplementary Material

## Supplementary Figures


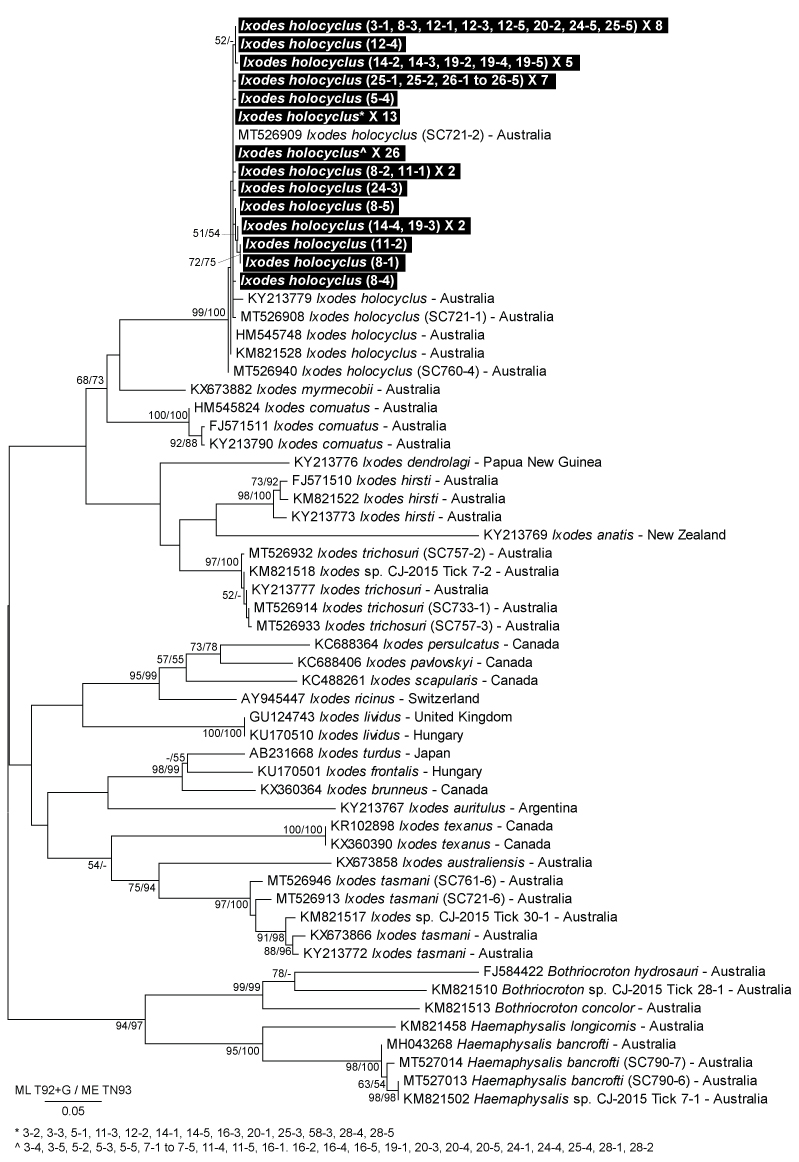


**Supplementary Figure 1.** Molecular phylogenetic analysis of *I. holocyclus* at the *cox*1 mitochondrial DNA marker. Codon positions included were 1st and 2nd. Bootstrap support values (1000 replications) are shown on appropriate nodes. The bootstrap confidence intervals (%) have been grouped as follows: ML T92+G / ME TN93, and only values >50% are shown.


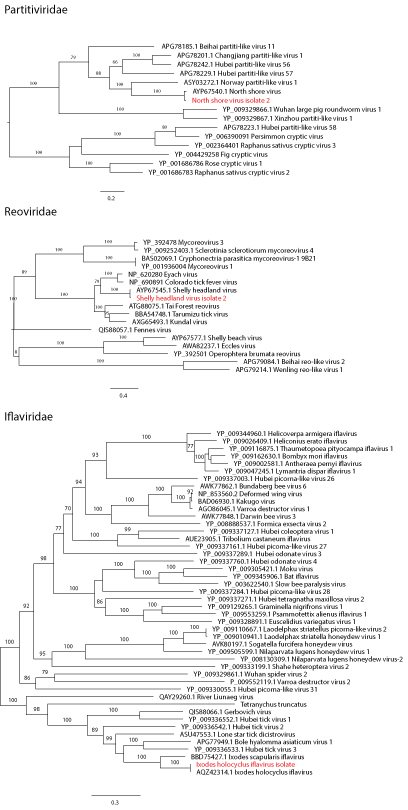


**Supplementary Figure 2.** Maximum likelihood phylogenetic trees showing the three novel virus sequences identified in this study from diverse families of RNA viruses. The trees are midpoint rooted for clarity and bootstrap values of over 65% are shown. The names of viruses identified in this study are shown in red text.

**Supplementary Table 1.** Species of fungi and eukaryotic microbes detected in the tick pooled libraries, which were previously associated with disease in mammals.

| Hit | Loc03 | Loc05 | Loc07 | Loc08 | Loc11 | Loc12 | Loc14 | Loc16 | Loc19 | Loc20 | Loc24 | Loc25 | Loc26 | Loc28 |
| --- | --- | --- | --- | --- | --- | --- | --- | --- | --- | --- | --- | --- | --- | --- |
| *Acremonium sclerotigenum* |  |  |  |  |  |  |  |  | X |  |  |  |  |  |
| *Aspergillus ochraceus* |  | X |  | X | X |  |  |  |  |  |  |  |  |  |
| *Beauveria bassiana* |  |  |  |  |  |  |  |  |  |  |  |  |  | X |
| *Besnoitia jellisoni* |  |  |  |  | X |  |  | X |  |  |  |  |  |  |
| *Brugia malayi* |  |  |  |  |  |  |  |  | X | X |  |  |  |  |
| *Conidiobolus coronatus* |  |  |  |  |  |  | X |  |  |  | X | X |  |  |
| *Conidiobolus lamprauges* | X |  |  |  |  |  |  |  |  |  |  | X |  |  |
| *Geomyces* sp. |  |  |  |  |  |  | X |  |  |  |  |  |  |  |
| *Histoplasma capsulatum* |  |  |  | X |  |  |  |  |  |  |  |  |  |  |
| *Malassezia globosa* |  | X |  | X |  |  |  |  |  |  |  |  |  |  |
| *Meyerozyma guilliermondii* |  |  |  |  |  |  | X |  |  |  |  | X | X |  |
| *Microsporum audouinii* |  |  |  |  |  |  |  |  |  |  |  |  | X |  |
| *Parastrongyloides trichosuri* |  | X |  |  |  |  |  | X |  |  |  |  |  |  |
| *Pseudogymnoascus pannorum* |  |  |  |  | X |  | X |  |  |  |  |  |  |  |
| *Roussoella solani* |  |  |  |  |  |  |  |  |  |  |  | X |  |  |
| *Schistosoma japonicum* |  |  | X | X | X |  | X |  |  | X |  |  |  | X |
| *Schistosoma mansoni* |  |  | X |  |  |  |  |  |  |  |  |  |  |  |
| *Spirometra erinaceieuropaei* |  |  |  |  |  |  |  |  |  |  |  | X |  |  |
| *Strongyloides papillosus* |  |  |  |  |  |  | X |  | X |  |  |  |  |  |
| *Wickerhamomyces anomalus* |  |  |  |  |  |  | X |  |  |  |  |  |  |  |
